# Precision Medicine in Rare Diseases

**DOI:** 10.3390/diseases8040042

**Published:** 2020-11-13

**Authors:** Irene Villalón-García, Mónica Álvarez-Córdoba, Juan Miguel Suárez-Rivero, Suleva Povea-Cabello, Marta Talaverón-Rey, Alejandra Suárez-Carrillo, Manuel Munuera-Cabeza, José Antonio Sánchez-Alcázar

**Affiliations:** Centro Andaluz de Biología del Desarrollo (CABD-CSIC-Universidad Pablo de Olavide), and Centro de Investigación Biomédica en Red: Enfermedades Raras, Instituto de Salud Carlos III, 41013 Sevilla, Spain; villalon.irene@gmail.com (I.V.-G.); monikalvarez11@hotmail.com (M.Á-C.); juasuariv@gmail.com (J.M.S.-R.); sulevapovea@gmail.com (S.P.-C.); martatalrey@gmail.com (M.T.-R.); asuacar1@gmail.com (A.S.-C.); mmuncab@upo.es (M.M.-C.)

**Keywords:** precision medicine, rare diseases, neurodegeneration with brain iron accumulation, mitochondrial diseases, congenital myopathies

## Abstract

Rare diseases are those that have a low prevalence in the population (less than 5 individuals per 10,000 inhabitants). However, infrequent pathologies affect a large number of people, since according to the World Health Organization (WHO), there are about 7000 rare diseases that affect 7% of the world’s population. Many patients with rare diseases have suffered the consequences of what is called the diagnostic odyssey, that is, extensive and prolonged serial tests and clinical visits, sometimes for many years, all with the hope of identifying the etiology of their disease. For patients with rare diseases, obtaining the genetic diagnosis can mean the end of the diagnostic odyssey, and the beginning of another, the therapeutic odyssey. This scenario is especially challenging for the scientific community, since more than 90% of rare diseases do not currently have an effective treatment. This therapeutic failure in rare diseases means that new approaches are necessary. Our research group proposes that the use of precision or personalized medicine techniques can be an alternative to find potential therapies in these diseases. To this end, we propose that patients’ own cells can be used to carry out personalized pharmacological screening for the identification of potential treatments.

## 1. Precision Medicine in Rare Diseases

Despite the important emphasis placed on the investigation of rare diseases and the development of orphan drugs by national governments, the pharmaceutical industry and private foundations, there are no adequate treatments for approximately 95% of rare diseases. The advance of genomics and functional proteomics has placed medicine in recent years at the gates of a new revolution, precision medicine, characterized by the development of molecular, genetic and cellular therapies that materialize in specific treatments for particular patients [1]. The rationale for this approach is that different mutations and inter-individual genetic variability can influence significantly both the disease sensitivity and the response to particular pharmacological therapies. The goal of personalized medicine is to maximize the likelihood of therapeutic efficacy and minimize the risk of drug toxicity for an individual patient.

The last decade has witnessed a rapid acceleration in our understanding of the genetic basis of many diseases. With this greater understanding comes the possibility of redefining the disease at a higher resolution and, along with this, aiming for a more precise therapy.

The precision medicine strategy has been successfully applied in different areas of medical care such as cardiology, oncology and nutrition and has a hopeful future in rare diseases [2].

## 2. Fibroblast Cultures and Transdifferentiation

**Figure 1:**
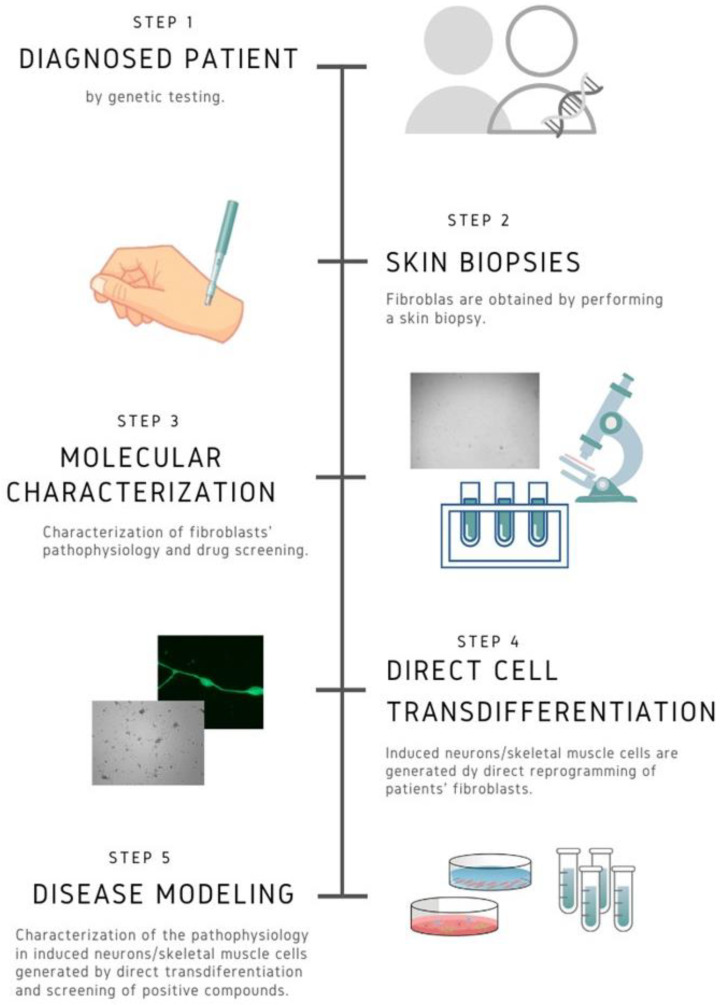
Work flow in personalized medicine. Fibroblasts and induced neurons or skeletal muscle cells derived from patients allow the characterization of the pathophysiological alterations in the patient’s own cells and in the most affected cell types. This approach gives the opportunity of evaluating personalized treatment options, taking into account the particular mutation and the genetic background of the patient.

At present, fibroblasts cell cultures derived from patients are easily obtained by means of skin biopsies. These cellular models are very informative to understand the pathophysiological alterations and the response of particular mutations to specific treatments. Furthermore, current direct or indirect cell transdifferentiation techniques make it possible to generate two of the most affected cell types in many rare diseases such as neurons or muscle fibers [3,4]. This personalized approach can be useful both for the evaluation of new drugs and for the repositioning of existing ones (Figure 1). Another advantage of precision personalized medicine is that it allows the evaluation of cellular response to combinations of different drugs, thereby diversifying possible therapeutic targets and optimizing potential treatments for patients.

### 2.1. Two Transdifferentiation Approaches (Direct vs. Indirect): Advantages and Drawbacks

#### 2.1.1. Human Induced Pluripotent Stem Cells (iPSCs) Model

In 2006, a hallmark publication by Yamanaka et al. redefined the field of stem cell biology [5]. For the first time, adult fully differentiated somatic cells were dedifferentiated to the pluripotent state using four transcription factors; *OCT4* (also known as *POU5F1*), *SOX2*, *KLF4* and *MYC*, yielding iPSCs, which have the capacity to differentiate into all types of somatic cells. These stem cell models carrying patient-specific mutations have become a valuable tool for rare disease research [3]. iPSCs provide numerous advantages as models for biomedical research. First, inducing pluripotency from somatic cells offers a non-invasive method to obtain cellular models for particular mutations and the generation of certain cell types that are difficult to obtain directly from humans. Second, iPSC models enable a sufficient number of cells for experimental work [6]. Thus, this model has been used for pathomechanism studies, differentiation assays, and drug screening [7].

However, the use of iPSCs for disease modelling has several disadvantages, such as its complexity and high cost of production [8]. Moreover, induction of pluripotency is accompanied by epigenetic reprogramming and a metabolic shift from OXPHOS toward glycolysis and vice versa during differentiation. Furthermore, the high expression of pluripotency genes increases the number of tumorigenic cells remaining in culture after differentiation [9]. In addition, it has been reported that the nuclear genome of iPSCs is genetically instable and frequently harbours mitochondrial DNA (mtDNA) aberrations [10]. For instance, it has been reported in inherited mitochondrial diseases that nuclear reprogramming reduces the copy number of mtDNA and may change the proportions of wild-type and mutant mtDNA (the degree of heteroplasmy), which determines the onset and severity of the symptoms of mitochondrial diseases. In addition, the degree of mtDNA heteroplasmy has been suggested to vary among different iPSC clones, indicating uneven mitochondrial segregation during reprogramming. Therefore, it is possible to obtain mtDNA mutation-free clones of iPSCs from patients with pathogenic mtDNA mutations. However, this fact can be an advantage since patient-derived mutation-free clones of iPSCs could be used for cell replacement therapies.

#### 2.1.2. Direct Reprogramming

Since the first direct reprogramming of a differentiated cell type into another was achieved, it took more than twenty years to reprogram fibroblasts into neuronal cells [11]. The first successful direct conversion of murine fibroblasts into induced neurons (iNs) was achieved in 2010, when Wernig and colleagues identified a combination of three proneural factors (the proneural gene ASCL1 and the transcription factors Brain-2 [BRN2] and Myelin transcription factor 1 like [MYT1L]), which were able to convert murine embryonic and postnatal fibroblasts into functional neurons in vitro [12]. The expression of these three transcription factors was required to obtain electrophysiologically functional neurons. Since then, iNs are defined as the product of directly reprogrammed neurons starting from somatic cells avoiding passing through the pluripotent stage. About one year later, this approach was transferred to human fibroblasts, using the additional factor Neurogenic Differentiation factor 1 (NEUROD1) to obtain iNs [13]. It was observed that longer conversion times were needed for human cells in comparison to mouse cells. Since then, the strategy of solely expression of different transcription factors has been successfully used to generate iNs [14,15,16]. However, from the beginning, the generation of iNs using direct reprogramming has had a main challenge: reaching a high conversion efficiency, which is defined as the percentage of iNs obtained relative to the number of starting cells plated. Although it can be very variable depending on the starting cells and the protocol used, the first approaches using direct reprogramming obtained very poor conversion efficiencies [17,18]. Moreover, it is also necessary to reach a good purity percentage, which is defined as the number of iNs in the final population related to the total cells in the plate. These two parameters are crucial since neurons are post-mitotic cells not able to further expand. In conclusion, a main drawback of direct transdifferentiation is a limited number of functional cells that can be obtained, which hence unmeet the requisite for drug screening [19]. Over the next years, new tools and strategies have been found to improve efficiency and purity of neuronal conversion. For example, the expression of different combinations of neuronal-specific miRNAs has been shown to direct the conversion of somatic cells into iNs [20,21], although most times the iNs generated were immature and conversion efficiencies were low.

Apart from using miRNAs, it has been demonstrated that derepression of neural genes can be a different approach to neural direct conversion. For example, repression of RNA-binding polypyrimidine tract-binding (PTB) protein enabled the miRNA action on several components of the REST (RE1-silencing transcription factor) complex and the subsequent expression of neuronal-specific genes [22]. REST is considered a relevant guardian for reprogramming, since it is expressed in non-neuronal cells where it represses neuronal genes [23]. A few years ago, it was shown that combinations of small molecules inhibiting both glycogen synthase kinase-3β and SMAD signalling together with the expression of neurogenic factors could improve conversion efficiency [24]. In fact, recently it was demonstrated that it is possible to undergo direct reprogramming into iNs by only chemically manipulating pathways involved in neural differentiation [25]. Currently, the combination of the different strategies seems to be the best approach to achieve high conversion efficiencies during iNs generation. For example, a combination of proneural genes overexpression (*ASCL1*, *MYT1L* and *NEUROD1*) with miRNAs such as miR9/9* or miR124 resulted in the generation of functional neurons with mature physiological properties from human fibroblasts [18].

Combinations of small molecules together with the overexpression of proneural factors have successfully converted adult somatic cells into iNs [26,27]. Some strategies have combined small molecules, transcription factors and miRNAs expression [28] or repression of p53, hypoxic conditions and expression of proneural transcription factors [29].

### 2.2. Advantages and Disadvantages of Direct Reprogramming

Direct reprogramming has several advantages in comparison with the generation of iPSCs-derived cells, such as the relative simplicity and short time requirements; thus, the cost in a clinical setting is reduced [17]. In addition, iNs, unlike iPSCs, maintain the ageing [30] and the epigenetic marks of the donor [31,32], making them excellent candidates for modelling neuronal pathophysiology in age-related disorders. Moreover, unlike iPSCs that are susceptible to form tumors following transplantation [33], it has been demonstrated that iNs obtained through in vivo reprogramming do not cause tumorigenic processes. Therefore, in the future they could be a promising tool for cellular therapy, for example, by performing direct reprogramming in vivo of endogenous murine astrocytes or transplanted human cells [34]. For that reason, the generation of iNs or other cell types by direct reprogramming from patient-derived fibroblasts suffering from genetic rare diseases holds enormous promise for understanding the pathogenesis of these disorders and for discovering new therapy approaches. Meanwhile, proliferating cells are preferred to perform iPSCs reprogramming [35,36]; proliferation is neither a prerequisite nor an advantage for direct neuronal conversion [37], which is important to directly convert senescent patient-derived cells.

Nevertheless, it remains unknown whether patient-derived fibroblasts can be directly reprogrammed into iNs that reflect the main pathological features of genetic rare diseases. Moreover, the retroviral or lentiviral vectors commonly used to introduce transcription factors are potentially dangerous to cause undesired genetic mutations. Specifically, iNs have their own challenges as an in vitro model to study diseases pathophysiology. First, in vitro expansion and passaging of cells prior to reprogramming prevents successful conversion [38,39]. Moreover, maintaining iNs in culture is difficult and achieving a great number of iNs during long-term cultures is very challenging, restrictive and expensive, since cell death can be observed from 30 days post-infection (DPI). This fact may hinder electrophysiological characterization of iNs, since the earliest time point when spontaneous action potentials could be detected to date in human iNs is 46 DPI [29] and they are usually observed at 80–100 DPI [40]. Moreover, iNs tend to form clusters during the reprogramming process, hampering isolation of individual cells for further analysis.

### 2.3. Applications of Direct Reprogramming

Direct transdifferentiation holds great promise for biomedical applications such as regenerative medicine and cell-based disease modelling. Somatic cells (for example, skin fibroblasts) can be directly converted into other tissue-specific cell types. The converted cells can be used in disease modelling (to analyze a disease-specific phenotype in a disease-associated somatic cell type in vitro) [25,41,42,43,44,45,46], pharmacological screening (to identify drugs interfering with the disease phenotype), drug toxicity testing (to identify side effects of potential drugs in different somatic cell types) and cell replacement therapy [47], since it is possible to direct convert cells in vivo, avoiding the need of immunosuppression.

Currently, the cell therapy approach best studied and closest to clinical application is transplantation of fetal progenitor cells of the neuronal subtype affected in the disease [48,49]. However, in the last years, several investigations have been focused on in vivo neural reprogramming from astrocytes or oligodendrocyte progenitors/NG2 glia [50].

It is important to realize that in vivo reprogramming not only takes place in a damaged and non-controlled environment, but also in a complex mixture of many different cell types. The first attempt was turning reactive glial cells into neurons after brain injury a few years ago [51]. Since then, great advances have been made regarding the efficiency of conversion and maturity of iNs [52]. Many cell types in several different brain regions have been targeted by various neurogenic fate determinants with the use of diverse viral vectors. For example, direct conversion of astrocytes into iNs in the striatum has been achieved using different reprogramming factors combinations, such as ASCL1/BRN2/MYT1L [34], SOX2 [53,54] or NEUROD1 [55]. Moreover, direct conversion from astrocytes has been achieved in the cortex [56] and the spinal cord [57,58]. Using NG2-glia as starting cells, direct neural conversion has been achieved using similar reprogramming factors (NEUROD1, SOX2) in the spinal cord [58] and cortex [56,59]. Other combinations of transcription factors (NEUROG2 and/or BCL2) have promoted the neural conversion from NG2-glia in striatum [60] and cortex [61]. The potential clinical application of direct reprogramming in vivo is increasingly closer. Encouragingly, in vivo direct conversion of dopaminergic neurons from striatal astrocytes has been recently achieved, partially recovering the function and behavior in a Parkinson’s disease (PD) mouse model [62]. Although this approach is still under development, it represents the main strategy to exploit the potential of direct neuronal reprogramming.

## 3. Braincure/Mitocure/Myocure Platforms

Our research group has developed three platforms for performing precision or personalized medicine in rare diseases: Braincure platform for rare neurodegenerative diseases with brain iron accumulation; Mitocure platform for mitochondrial diseases; and Myocure platform for congenital myopathies.

### 3.1. Braincure Platform

Neurodegeneration with brain iron accumulation (NBIA) is a group of rare neurodegenerative disorders of genetic origin, characterized by a dysfunction of the central nervous system and the accumulation of iron in certain areas of the brain that causes the progressive disability of patients [63]. Most NBIAs begin clinically in childhood and are inherited with an autosomal recessive pattern. Currently, there are no effective treatments for the great majority of these diseases.

The objective of this platform is to deepen the pathophysiology of the disease and find effective personalized treatments using fibroblasts and neuronal cells derived from NBIA patients. For this, we characterize the pathophysiological mechanisms and evaluate the effectiveness of a library of commercial pharmacological compounds in the recovery of pathological alterations in the patient-derived cells.

In a first stage, pharmacological screening is carried out in fibroblasts derived from NBIA patients. For high throughput screening, a library of 426 drugs approved by the U.S. FDA (United States Food Drug Administration) from Selleck Chemicals (Houston, TX, USA) including a selection of compounds frequently used in NBIA patients treatment (iron chelators, antioxidants, pantothenate, creatine etc.) are routinely evaluated. As iron accumulation can be easily detected in fibroblasts derived from NBIA patients by Perls’ Prussian blue reaction [64], drug hits are identified by a >75% reduction of intracellular iron accumulation. To confirm the defect rescue in NBIA mutant fibroblasts, in deep evaluation of iron metabolism alterations, expression levels of mutant enzymes, mitochondrial dysfunction, oxidative stress, lipid metabolism and spontaneous and induced apoptosis are examined in NBIA fibroblasts after treatment with selected favorable compounds. In parallel, INs are generated by direct reprograming from patient fibroblasts. The most favorable compounds in fibroblasts screening are selected for testing in mutant iNs.

#### 3.1.1. Strategy for the Identification of Effective Treatments for Neurodegeneration Associated with Pantothenate Kinase (PKAN)

The most prevalent NBIA subtype is the neurodegeneration associated with pantothenate kinase (PKAN) due to mutations in the enzyme pantothenate kinase 2 (PANK2), which participates in the first reaction of the coenzyme A biosynthesis pathway. From the pathophysiological point of view, these mutations cause deficiency of coenzyme A, accumulation of iron and lipofuscin and a marked increase in oxidative stress. Mutations also cause low levels of expression of the mutant enzyme [65]. Our findings indicate that pantothenate treatment upregulates protein expression levels of PANK2 in fibroblasts obtained from patients carrying particular mutations. In addition, we have confirmed that pantothenate had also a favorable effect in iNs produced by direct reprogramming of patient skin cells. The restoration of PANK2 expression levels was accompanied by the correction of all the pathophysiological alterations such as coenzyme A deficiency, iron and lipofuscin accumulation, bioenergetics failure and increased oxidative stress [65]. These results suggest that pharmacological screening in cellular models can be a useful tool to identify PANK2 mutations with residual enzymatic activity that respond to pantothenate supplementation. Even more important, the existence of PANK2 residual expression that can be significantly corrected in cells obtained from patients points out to the possibility of an effective treatment with pantothenate at high doses. This hypothesis must be corroborated by comparing both the effect of pantothenate in vitro and in patients in controlled clinical trials. The knowledge of the multiple types of PANK2 gene mutations and their response to pantothenate supplementation gives new opportunities for the implementation of precision personalized therapies in PKAN.

In addition, these personalized detection strategies in PKAN can facilitate the identification of new pharmacological chaperones (PC) able to restore the expression levels and activity of the dysfunctional enzyme. Preliminary results from our group have detected several commercial drugs, which are able to correct PANK2 expression levels as well as most of the physiopathological alterations in fibroblasts derived from PKAN patients.

A large number of mutations related to human diseases cause the destabilization of specific proteins. Interestingly, molecules that function as PC can rescue the activity of unstable proteins [66,67,68]. However, in a specific disorder, PC therapy will be adequate depending on its genotype [69]. Corroborating this hypothesis, our findings have shown that many mutations in PANK2, but not all, can respond positively to pantothenate supplementation [65,70]. Therefore, a strategy to identify more PCs capable of correcting PANK2 expression and activity in cells such as fibroblasts or/and induced neurons can lead to potential treatments in particular patients. Following this strategy, several drugs have been already repositioned as a PC for rare diseases treatment [71]: doxorubicin, an antitumor anthracycline, for cystic fibrosis [72]; diltiazem, an antihypertensive, for Gaucher’s disease [73]; ambroxol, a mucolytic agent, for Fabry and Gaucher disease [74]; acetylcysteine, another mucolytic agent, for Pompe disease [75]; pyrimethamine, an antiparasitic drug, for GM2 gangliosidosis [76]; carbamazepine, a dibenzazepine, for hyperinsulinemic hypoglycemia [77]; and salicylate, a known anti-inflammatory, for Pendred syndrome [78]. Recently, a PC allosteric activator of PANK (PZ-2891) has been identified [79]. Interestingly, PZ-2891 crosses the blood-brain barrier and improves the phenotype in a mouse model with cerebral coenzyme A deficiency.

#### 3.1.2. Precision Medicine in PKAN

The implementation of precision personalized medicine for the identification of potential treatment of neurodegenerative disorders such as PKAN seems to be very promising in contrast to the traditional “single drug for all patients” strategy [80]. In fact, neurodegenerative disorders can have variable clinical characteristics even in patients with the same mutation; therefore, it is very unlikely that all the patients will respond to a single drug. In this context, a precision medicine approach using fibroblasts and iNs derived from patients with PKAN may represent an attractive opportunity to find effective treatments.

Braincure platform performs precision medicine in the most prevalent NBIA disorders (Table 1): PKAN, pantothenate kinase-associated neurodegeneration, caused by mutations in the PANK2 gene; PLAN, PLA2G6-associated neurodegeneration, due to mutations in the PLA2G6 gene; BPAN, Beta-propeller protein-associated neurodegeneration, caused by mutations in the WDR45 gene; MPAN, Mitochondrial-membrane Protein-associated Neurodegeneration, due to mutations in the C19orf12 gene; and FHAN, Fatty acid hydroxylase-associated neurodegeneration, caused by mutations in the FAH2 gene). We are currently performing personalized medicine in more than 50 patients from both Spain and abroad (Brazil, Colombia, Mexico, USA, France, United Kingdom, Holland, Hungary and Poland).

### 3.2. Mitocure Platform

Mitochondrial diseases include a group of chronic and progressive muscular and neurodegenerative disorders caused by a great variety of mutations in nuclear (nDNA) or mitochondrial DNA (mtDNA), most of which have no effective treatment [81]. These diseases have a great heterogeneity and fundamentally affect the energy production capacity of the cells.

Current pharmacological therapies are based primarily on: (1) Eliminate toxic metabolites; (2) Try to circumvent the blockages of the respiratory chain; (3) Administer metabolites and cofactors to improve the synthesis of ATP; and (4) Prevent oxidative stress [82].

Given the diversity of mutations and the different therapeutic options, our proposal argues that a personalized therapeutic approach is required in mitochondrial diseases. In the Mitocure platform, we evaluate the therapeutic effectiveness of currently available treatments in fibroblasts derived from mitochondrial patients and in neuronal cells generated by direct reprogramming. To achieve this objective, we study the effects of these treatments on the pathophysiological alterations present in mutant cells. As a screening strategy, we examined cell proliferation and/or cell death in galactose culture medium (which forces energy to be obtained by the mitochondria) [83,84], using an automated platform for live-cell imaging (Celldiscoverer 7, Zeiss). Combined analyses of phase-contrast and fluorescence images allow assessment of treatment effects on cell proliferation as well as the extent and kinetics of cell death. A pharmacology library of compounds frequently used in the treatment of mitochondrial patients is evaluated: antioxidants, AMP-activated protein kinase (AMPK) activators, autophagy/mitophagy modulators, mitochondrial dynamics modulators; inflammasome inhibitors; mitochondrial unfolded protein response (UPR^mt^) activators; and combinations of several treatments.

Next, positive compounds are confirmed by assessing their effect on mitochondrial respiratory chain activity, expression levels of mitochondrial proteins, mitochondrial membrane potential, oxidative stress, and the activation of mitophagy and/or apoptosis in fibroblast and iNs. The relevance of cellular models derived from patients with inherited mitochondrial disorders for pathomechanistic studies and evaluation of therapies have been previously explained by the group of Ann Saada [85,86]. Currently, Mitocure platform is performing precision measurement in more than 20 mutations that directly affect mitochondrial oxidative phosphorylation (Table 2).

#### Mitocure-KAT6A Platform

Intellectual disability (ID) or global developmental delay (GDPR) occurs in 1–3% of all children. Variations in the number of copies of genes and rare mutations (often linked to the X chromosome or autosomal recessive) explain up to 25% of all cases. With the development of massive DNA sequencing techniques, numerous heterozygous monogenic mutations have emerged as the leading cause of different intellectual disability syndromes and are responsible for up to 40% of severe ID mutations [87,88]. Mutations in several genes involved in epigenetic regulation of gene expression have been linked to different ID syndromes including alterations of the lysine-acetyltransferase gene in KAT6A syndrome [89].

Lysine-acetyltransferase 6A (KAT6A) belongs to the MYST family of histone acetyltransferases that are defined by the presence of a highly conserved MYST domain consisting of a motif of binding to acetyl-CoA and a zinc finger [90]. The MYST family of proteins (KAT6A, KAT6B, KAT5 and KAT7) participate in a wide range of central cellular functions, such as chromatin remodeling, gene regulation, protein translation, metabolism and cell replication [91].

Most of the clinical features in KAT6A syndrome have a very variable penetration. The basic pathological characteristics are microcephaly, intellectual disability, speech delay, cardiac alterations and gastrointestinal complications [92].

Recent hypotheses (Dr. Richard I. Kelley, Kennedy Krieger Institute, Department of Pediatrics, Johns Hopkins Medical Institutions, http://mitomedical.com/research/) point out that mutations in the KAT6A gene affect secondary mitochondrial function and that drugs that act at the mitochondrial level (such as carnitine and vitamin B5) are capable of reversing the clinical phenotypes of the disease [93].

In the Mitocure-KAT6A platform, we evaluate the therapeutic effectiveness of several commercially available treatments that act at the mitochondrial level in fibroblasts derived from KAT6A patients and in neuronal cells generated by direct reprogramming. To achieve this objective, we study the effects of these treatments on the pathophysiological alterations present at the cellular level such as cell proliferation, mitochondrial respiratory chain enzymatic activities, coenzyme Q_10_ levels, mitochondrial protein expression levels, mitochondrial membrane potential, and activation of mitophagy and/or apoptosis.

### 3.3. Myocure Platform

Congenital myopathies are a group of genetic muscle diseases that are classified based on the histopathological characteristics observed in muscle biopsy [94]. They are subdivided by the predominant structural pathological change on muscle biopsy, resulting in five subgroups [95]: (1) core myopathies; (2) nemaline myopathies; (3) centronuclear myopathies; (4) congenital fiber-type disproportion myopathy; and (5) myosin storage myopathy. To date, only supportive treatments are available.

Nemaline myopathy (NM), which is part of this group of diseases, was described for the first time as a non-progressive congenital musculoskeletal disorder, characterized by the presence of inclusions in muscle fibers called “nemalinic rods” [96]. This pathology contains a wide genetic heterogeneity that produces a similar phenotype [97]. Its incidence is 1 in 50,000 live births; although in our country the incidence of this pathology is unknown, since in clinical practice it is a rare disease [98]. Mutations have been found in more than 10 different genes that cause the disease; seven of which code for sarcomeric thin filament components (NEB, ACTA1, TPM2, TPM3, TNNT1, CFL2, LMOD3) and 3 genes (KBTBD13, KLHL40 and KLHL41), belonging to the BTB-BACK-kelch family of proteins (BBK) involved in the ubiquitin-proteasome pathway [99].

The objectives of the Myocure platform are: (1) To establish cellular models to understand the pathophysiological mechanisms of NM. (2) To identify potential therapies by developing a pharmacological screening methodology in cell models of NM.

As a screening strategy, we assess the correct formation of actin filaments by fluorescence microscopy techniques using the CellDiscoverer7 Image Analysis platform (Zeiss). Positive compounds are those that are capable of restoring the correct formation of actin filaments. Next, favorable compounds are confirmed by verifying the improvement of all pathophysiological alterations. A collection of compounds are routinely tested; phalloidin (stabilizer of actin filaments); several combinations of amino acids (tyrosine, carnitine, taurine, or creatine); salbutamol (selective ß2-adrenergic agonist of bronchial smooth muscle); Ras homolog family member A (Rho) stimulators (forskolin); Rho-associated, coiled-coil containing protein kinase (ROCK) inhibitors (Y-27632 2HCl); myostatin inhibitors (anti-myostatin antibodies, ACE-031); follistatin activators (natural myostatin inhibitor); UPR stimulators: nicotinamide.

Currently, the Myocure platform is performing precision medicine in 8 patients with nemaline myopathy (Table 3). In a second phase, if the results are positive in the pharmacological screening in fibroblasts, we will confirm the positive findings in skeletal muscle cells generated by direct reprogramming of patients’ fibroblasts [100].

This mutation-specific therapeutic approach can potentially be applied for other hereditary muscular diseases such as Duchenne muscular dystrophies (DMD) and facioscapulohumeral muscular dystrophy. In the particular case of DMD, as molecular treatments aimed at dystrophin restoration are increasingly available as commercialized drugs or within clinical trials, genetic diagnosis has become an indispensable tool in order to determine eligibility for specific treatments [101]. Thus, DMD patients harboring deletions in exon 44, 45, 51 or 53 may be eligible for inclusion in one of several ongoing clinical trials of exon skipping. Patients harboring nonsense mutations that cause the synthesis of the dystrophin protein to stop prematurely may be eligible for treatment with ataluren, which promotes ribosomal read-through of premature stop codons [101]. It is reasonable to propose that particular mutations should be also evaluated at the cellular level and confirm in vitro the effectiveness of potential treatments to minimize the frequency of non-responder patients.

## 4. Conclusions

The own patient-derived cells can be used to perform personalized pharmacological screening in genetic rare diseases. For precision medicine to be successful at the therapeutic level, in addition to the information provided from genomics, pharmacogenomics, metabolomics and proteomics, our proposal argues that it is also necessary to know the cellular response, and therefore the behavior of particular mutations in vitro, to various therapeutic options. Precision medicine relies on the assumption that different mutations and marked inter-individual genetic variation can contribute significantly to drug response. The goal of personalized medicine is to maximize the probability of therapeutic efficacy for an individual patient.

## Figures and Tables

**Table 1:** Neurodegeneration with brain iron accumulation (NBIA) subtypes under study in the Braincure platform.

NBIA Subtype	Gene	Chromosome Location	Biological Process	Associated Diseases
Pantothenate kinase-associated neurodegeneration (PKAN)	PANK2	20p13	Regulation of CoA biosynthesis	Neurodegeneration with brain iron accumulation 1 Classic PKAN Atypical PKAN HARP syndrome
Phospholipase A2 group VI-associated neurodegeneration (PLAN)	PLA2G6	22q13.1	Membrane remodeling	Neurodegeneration with brain iron accumulation 2 Infantile Neuroaxonal Dystrophy Atypical Neuroaxonal Dystrophy Parkinson disease 14
Mitochondrial-membrane proteins-associated neurodegeneration (MPAN)	C19ORF12	19q12	Apoptotic process Autophagy Mitochondrial calcium ion homeostasis Response to oxidative stress	Neurodegeneration with brain iron accumulation 4 Spastic paraplegia 43
Beta-propeller protein-associated neurodegeneration (BPAN)	WDR45	Xp11.23	Autophagy	Neurodegeneration with brain iron accumulation 5 Static encephalopathy of childhood with neurodegeneration in adulthood (SENDA)
Fatty acid hydrolase-associated neurodegeneration (FHAN)	FAG2H	16q23.1	Lipid biosynthesis	Spastic paraplegia 35 Leukodystrophy

**Table 2:** Genes implicated in mitochondrial diseases. Mitochondrial and nuclear DNA mutation-causing inherited mitochondrial disorders. The table presents the mutations that are under study in the Mitocure platform. mtDNA, mitochondrial DNA; nDNA, nuclear DNA.

Gene	Genome	Biological Process	Associated Diseases
*MT-ND1*	mtDNA	Electron transport	Leber hereditary optic neuropathy (LHON) Mitochondrial complex I deficiency, mitochondrial type 3 Leigh syndrome, MELAS syndrome Diabetes mellitus, non-insulin-dependent (NIDDM)
*MT-ND3*	mtDNA	Electron transport	Leigh syndrome Mitochondrial complex I deficiency, mitochondrial type 1 Parkinson disease
*NDUFS1*	nDNA	Electron transport	Mitochondrial complex I deficiency, nuclear type 5
*COX15*	nDNA	Electron transport Proton transmembrane transport Heme biosynthesis process	Mitochondrial complex IV deficiency, nuclear type 6
*GFM1*	nDNA	Protein biosynthesis	Combined oxidative phosphorylation deficiency 1
*OPA1*	nDNA	Apoptosis Sensory transduction Vision	Behr syndrome Optic atrophy 1 Optic atrophy plus syndrome Mitochondrial DNA depletion syndrome 14 (encephalocardiomyopathic type)
*LIPT01*	nDNA	Nitrogen compound metabolic process Protein modification process Lipid metabolic process	Lipoyltransferase 1 deficiency
*COQ7*	nDNA	Ubiquinone biosynthesis	Coenzyme Q_10_ deficiency, primary, 8
*NDUFV1*	nDNA	Electron transport	Mitochondrial complex I deficiency, nuclear type 4
*NDUFAF6*	nDNA	Mitochondrial respiratory chain complex I assembly	Mitochondrial complex I deficiency, nuclear type 17 Fanconi renotubular syndrome 5
*NDUFS4*	nDNA	Electron transport	Mitochondrial complex I deficiency, nuclear type 1

**Table 3:** Genes implicated in Nemalinic myopathy disease. Gene mutations that cause Nemaline myopathy. Currently, the Myocure platform has different lines under study with mutations in *NEB* and *ACTA1* genes.

Gene	Cytogenetic Location	Biological Process	Associated Diseases
*NEB*	2q23.3	Actin binding	Nemaline myopathy 2
*ACTA1*	1q42.13	Actin filament polymerization and assembly	Nemaline myopathy 3 Myopathy, congenital, with fiber-type disproportion 1 Myopathy, actin, congenital, with excess of thin myofilaments Myopathy, actin, congenital, with cores Myopathy, scapulohumeroperoneal
*TPM2*	9p13.3	Actin filament organization	Nemaline myopathy 4 Cap myopathy 2 Arthrogryposis, distal, type 2B4 Arthrogryposis, distal, type 1A
*TPM3*	1q21.3	Actin filament organization	Nemaline myopathy 1 Cap myopathy 1 Myopathy, congenital, with fiber-type disproportion
*TNNT1*	19q13.42	Muscle filament signaling	Nemaline myopathy 5
*CFL2*	14q13.1	Actin filament depolymerization	Nemaline myopathy 7
*LMOD3*	3p14.1	Actin filament organization	Nemaline myopathy 10
*KBTBD13*	15q22.31	Post-translational process modifications Ubiquitin-proteasome pathway	Nemaline myopathy 6
*KLHL40*	3p22.1	Ubiquitin conjugation pathway	Nemaline myopathy 8
*KLHL41*	2q31.1	Myofibril assembly Post-translational protein modification Ubiquitin-proteasome pathway	Nemaline myopathy 9
